# The neuronal Golgi in neural circuit formation and reorganization

**DOI:** 10.3389/fncir.2024.1504422

**Published:** 2024-12-05

**Authors:** Naoki Nakagawa

**Affiliations:** ^1^Laboratory of Mammalian Neural Circuits, National Institute of Genetics, Mishima, Japan; ^2^Graduate Institute for Advanced Studies, SOKENDAI, Mishima, Japan

**Keywords:** brain development, neuronal maturation, neural circuit formation, neural circuit reorganization, Golgi apparatus, dendrite

## Abstract

The Golgi apparatus is a central hub in the intracellular secretory pathway. By positioning in the specific intracellular region and transporting materials to spatially restricted compartments, the Golgi apparatus contributes to the cell polarity establishment and morphological specification in diverse cell types. In neurons, the Golgi apparatus mediates several essential steps of initial neural circuit formation during early brain development, such as axon-dendrite polarization, neuronal migration, primary dendrite specification, and dendritic arbor elaboration. Moreover, neuronal activity-dependent remodeling of the Golgi structure enables morphological changes in neurons, which provides the cellular basis of circuit reorganization during postnatal critical period. In this review, I summarize recent findings illustrating the unique Golgi positioning and its developmental dynamics in various types of neurons. I also discuss the upstream regulators for the Golgi positioning in neurons, and functional roles of the Golgi in neural circuit formation and reorganization. Elucidating how Golgi apparatus sculpts neuronal connectivity would deepen our understanding of the cellular/molecular basis of neural circuit development and plasticity.

## 1 Introduction

**Figure 1:**
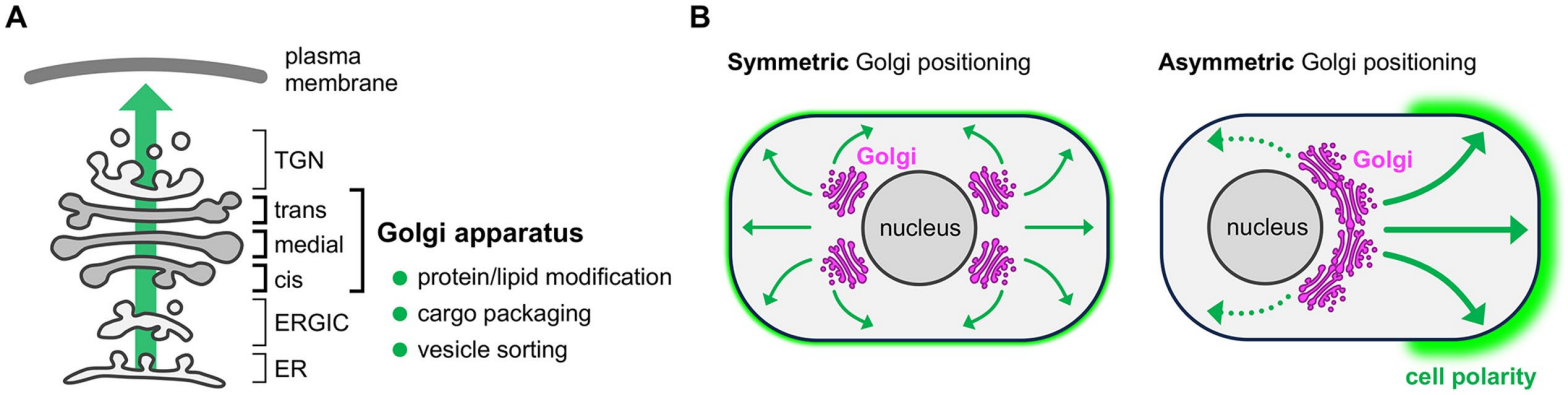
Functions of the Golgi apparatus and Golgi-mediated cell polarization. **(A)** Schematic of the intracellular secretory pathway. The Golgi apparatus is located at the center of membrane traffic, where newly synthesized proteins and lipids are modified and then packaged into vesicles for subsequent sorting to appropriate destinations via trans-Golgi network (TGN). ER, endoplasmic reticulum; ERGIC, ER-Golgi intermediate compartment. **(B)** Functional consequence of the Golgi positioning. Symmetric Golgi positioning within a cell leads to unbiased delivery of transport vesicles (indicated by arrows) and thus uniform plasma membrane composition throughout the cell (left). Asymmetric Golgi positioning within a cell leads to biased supply of vesicles (thick vs. dotted arrows) and thus uneven distribution of lipids and proteins, which generates a functionally distinct membrane domain and contributes to the cell polarity establishment (right, green shade).

The Golgi apparatus is a compartmentalized membranous organelle arranged in the cytoplasm as a stack of flattened cis-, medial-, and trans-Golgi cisternae (Figure 1A). In the intracellular secretory pathway, newly synthesized proteins and lipids in the endoplasmic reticulum (ER) are packaged into membrane carriers and exit the ER from ER exit sites (ERES). These molecular cargoes proceed to the pleiomorphic intermediate compartments connecting the ER and Golgi (ER-Golgi intermediate compartment, ERGIC) and then enter the Golgi apparatus through the cis-face of the Golgi stack (Saraste and Marie, 2018; Nakano, 2022). At the surface of the trans-most cisterna of the Golgi stack, there is a tubular and reticular structure termed the trans-Golgi network (TGN), which contains various budding carriers including clathrin-coated vesicles. Thus, TGN is thought to be the place of cargo sorting to send membrane carriers to appropriate subcellular destinations (Guo et al., 2014; Nakano, 2022). Lying as the central hub in the membrane trafficking, the Golgi apparatus plays a pivotal role in intracellular secretory pathway by modifying newly synthesized proteins and lipids, packaging molecular cargoes into secretory vesicles, and sorting vesicles to appropriate destinations (Figure 1A) (Guo et al., 2014; Boncompain and Weigel, 2018). Thus, positioning of the Golgi apparatus in the specific intracellular region can generate asymmetry in the membrane composition, and thereby contributing to the establishment of cell polarity in various cell types (Figure 1B) (Yadav and Linstedt, 2011; Ravichandran et al., 2020).

Accumulating evidence illustrates that in various brain regions, the Golgi-mediated cell polarity is critically involved in specifying and remodeling neuronal morphology. While acquisition of proper axonal and dendritic morphologies is essential for neural circuit formation, remodeling of these structures in response to the external stimuli is essential for adaptive circuit reorganization (Goodman and Shatz, 1993; Katz and Shatz, 1996). Therefore, clarifying the roles of the Golgi apparatus during neuronal maturation is a unique avenue to elucidate the cellular underpinnings of neural circuit development and plasticity.

**Figure 2:**
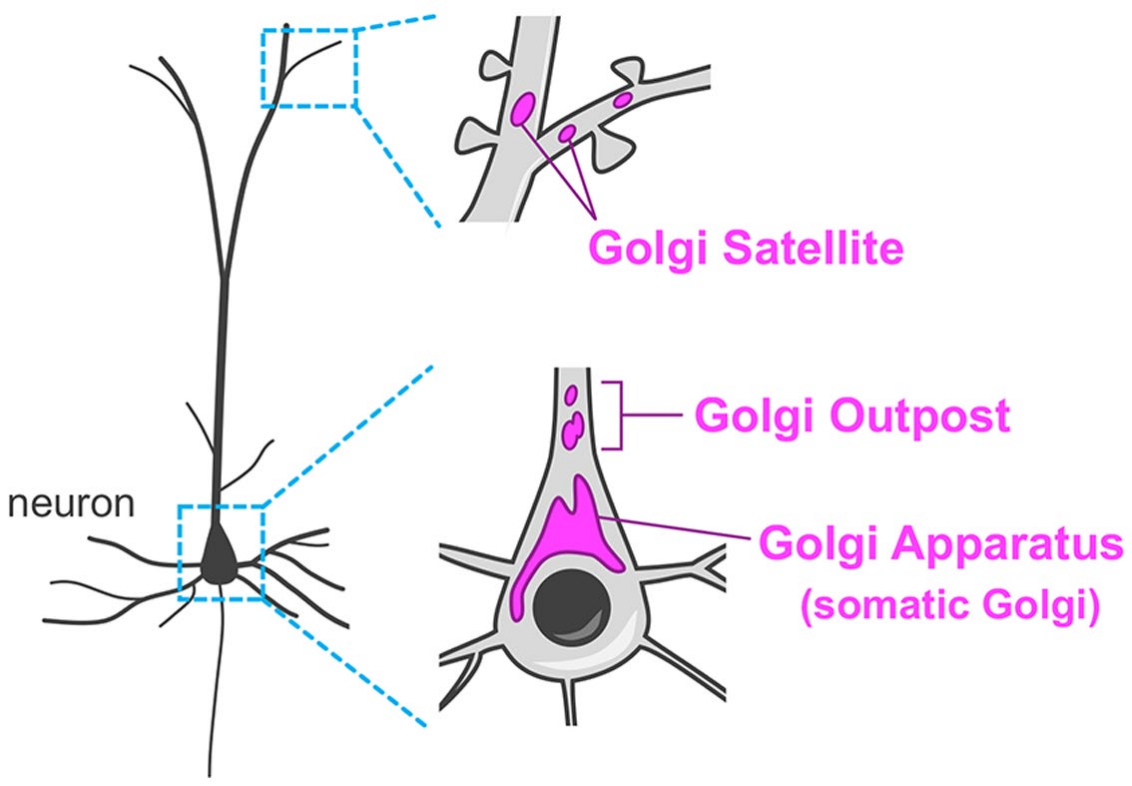
Organization of the Golgi apparatus in neurons. Neurons typically have the Golgi apparatus at the perinuclear region in their soma (somatic Golgi). In addition, neurons have remote Golgi stations. The Golgi outposts are usually found in the proximal region of the dendrites in mammalian neurons. The Golgi satellites are more widely distributed in dendrites including the distal segments.

Compared to other cell types in the body, neurons have extraordinarily complex morphology with multiple long and branched processes. Thus, their secretory system has enormous demands. For example, establishment of sophisticated neurite projection requires spatiotemporally coordinated membrane and molecular supply/withdrawal tailored to extension/retraction events in every axonal and dendritic branch. Another example is that distinct sets of molecules should be transported to all excitatory and inhibitory post-synaptic membranes. Neurons have revolutionized their secretory system by having remote Golgi units in dendrites (termed Golgi outposts and Golgi satellites), in addition to their “canonical” perinuclear Golgi apparatus (Figure 2) (Horton et al., 2005; Mikhaylova et al., 2016; Kennedy and Hanus, 2019; Wang et al., 2020). These unique distal Golgi stations contribute to effective trafficking and processing of cargoes in the highly demanding secretory system in neurons.

In this review, I summarize the characteristics of the Golgi apparatus positioning in various types of neurons and its dynamic changes during embryonic and early postnatal neuronal development. I also discuss the upstream regulators of Golgi positioning in neurons, and the roles of the Golgi apparatus in neuronal morphogenesis and functioning. I have arranged these topics in the order of developmental milestones observed during the different phases of neuronal maturation, including the axon-dendrite polarization, migration, primary dendrite specification, dendritic elaboration, activity-dependent postnatal dendritic remodeling, and the plastic response to excitatory stimuli. This will allow a chronological overview of the function, regulation, and developmental transition of the neuronal Golgi during the initial formation of neural circuits and its postnatal reorganization. I also discuss a potential relationship between the plasticity of the neuronal Golgi and neurological disease-associated alterations.

## 2 Golgi apparatus in neuronal polarization and migration

### 2.1 Determination of the axon-dendrite polarity

A neuron typically has a thin long axon and multiple relatively short but highly elaborate dendrites. Since axons and dendrites are the major sites of information transfer in neurons, it is critical for proper brain functioning that these neurites are appropriately projected to their target brain regions. Thus, determination of axon-dendrite polarity is controlled strictly by spatiotemporal mechanisms. Axon-dendrite polarity is determined in the early phase of neuronal maturation (Arimura and Kaibuchi, 2007; Barnes and Polleux, 2009). Extensive studies using cultured neurons have shown that neurons initially form multiple short neurites, and then become polarized when one of these neurites is specified as an axon (Dotti et al., 1988; Arimura and Kaibuchi, 2007; Barnes and Polleux, 2009). The remaining neurites are subsequently elaborated and become dendrites. Using the Ti1 neuron in peripheral nervous system of embryonic grasshopper as a model, Lefcort and Bentley described that the Golgi apparatus, labeled by either wheat germ agglutinin or NBD-ceramide, is located proximally to the prospective axon-initiation site (Lefcort and Bentley, 1989). Further, they found that the centrosome, tubulin, and actin filaments co-localized with Golgi apparatus in the axon-initiation site. Thus, the positioning of intracellular machineries and axon-initiation sites are spatially coupled. These *in vivo* results revealing the spatial relationships between the axon-initiation site and the Golgi apparatus positioning have been studied further using *in vitro* cultured neurons. In cultured mouse cerebellar granule cells and rat hippocampal neurons, the Golgi apparatus and the centrosome colocalize at the axon-initiation site (Zmuda and Rivas, 1998; de Anda et al., 2005). The cerebellar granule cells in culture show a multistep maturation, wherein they change from the unipolar stage with a single axon, to the bipolar stage with two axons before fully maturing to have the T-shaped parallel fiber axons and multiple dendrites (Powell et al., 1997; Consalez et al., 2021). Interestingly, the Golgi apparatus is first positioned at the base of the first axon during the unipolar stage, and then it is found at the base of the second axon during the bipolar stage (Zmuda and Rivas, 1998). In the fully matured granule cell, the Golgi position is no longer correlated to either axon or dendrite roots, indicating that the Golgi position is spatiotemporally regulated to support axon initiation during an appropriate time window.

Considering that Golgi apparatus modulates the directionality of intracellular vesicle transport, one may think that the Golgi contributes to axon formation via selectively supplying the membranes and proteins necessary for axonogenesis. Indeed, an earlier attempt using cultured hippocampal neurons has visualized the selective accumulation of post-Golgi vesicles (labeled by fluorescent-dye conjugated ceramide) in the neurite with the largest growth cone, which is destined to become an axon (Bradke and Dotti, 1997). Moreover, treatment with brefeldin A (BFA), an inhibitor of Arf GTPases that regulate secretory trafficking, impairs axon initiation and elongation (Jareb and Banker, 1997). The key materials contained in the transport vesicles, which drive axon initiation/elongation, are lipids and membrane proteins. Since neurite elongation requires massive expansion of membrane surface area, supply of the phospholipids derived either from *de novo* synthesis or intracellular membrane reservoirs seems to be the primary contributor of axonal growth (Lecuit and Pilot, 2003; Pfenninger, 2009). Sending proper sets of membrane proteins to the axonal tip is also important. Elongating axons navigate through the intricate extracellular space and eventually reach the target neurons to make synaptic connections, which stabilizes axonal structure. Therefore, receptors, adhesion proteins, and synaptic organizers necessary for axon guidance and synaptogenesis should be correctly expressed on the axonal tip (O’Donnell et al., 2009; Shen and Cowan, 2010). Excluding dendritic proteins from axonal compartment is also important, which is exemplified by the role of the protein kinase D (PKD). PKD is a protein kinase functioning at the Golgi apparatus. Silencing the PKD function causes mis-sorting of dendritic membrane receptors including transferrin receptor (TfR) and the low-density receptor-related protein (LRP) to the axon, which results in the failure of single axon specification and neuronal polarity establishment (Bisbal et al., 2008; Yin et al., 2008).

Subsequent research has identified molecular mechanisms regulating the membrane trafficking, which underlie axon formation and extension. The secretory pathway underlying axon development is composed of protein/lipid synthesis and modification at the ER and Golgi, vesicle budding from the Golgi, transport of the vesicles along axonal shaft, and vesicle fusion at the axonal growth cone (Pfenninger, 2009). A small GTPase Sar1, which regulates ER-to-Golgi vesicle transport, accumulates in axon and facilitates axonal growth (Aridor and Fish, 2009). Rab6 promotes transport of synaptic vesicle precursors from the Golgi to axon and thus regulates axon specification (Zhang et al., 2024). Axonal transport of the vesicles relies on microtubule-based mechanisms. For example, kinesin, a microtubule plus end-directed motor, is required for axon initiation and elongation (Ferreira et al., 1992; Deng et al., 2014). Other than the motor proteins, Rab family GTPases are involved. For instance, Rab10 is associated with plasmalemmal precursor vesicles and activated by Lgl1 (Wang et al., 2011). The activated Rab10 interacts with c-Jun N-terminal kinase-interacting protein 1 (JIP1), which, in turn, bind to kinesin-1 light chain, and thereby tethering the vesicles to the motor system (Deng et al., 2014). Knockdown of either Rab10, Lgl1, or JIP1 impairs neuronal polarity *in vivo* (Wang et al., 2011; Deng et al., 2014). Rab8 also regulates axon formation and elongation by promoting axonal transport *in vitro* (Huber et al., 1995). The last critical step is the vesicle fusion to the axonal membrane at the growth cone. The vesicle docking to the axonal membrane is regulated by insulin-like growth factor 1 receptor (IGF1R)-phosphatidylinositol 3-kinase (PI3K)-Akt pathway (Pfenninger et al., 2003; Laurino et al., 2005; Pfenninger, 2009). In addition, inhibition of the function of SNAP-25, one of the SNARE (soluble N-ethylmaleimide-sensitive-factor attachment receptor) complex proteins, reduces neurite extension, indicating the SNARE-based fusion machinery is required for the insertion of the vesicles to axonal membrane (Osen-Sand et al., 1993).

Altogether, these findings support that, during axon specification, post-Golgi vesicles are preferentially transported to the neurite destined to become the axon, where they supply materials for further axonal growth.

### 2.2 Control of the neuronal migration

**Figure 3:**
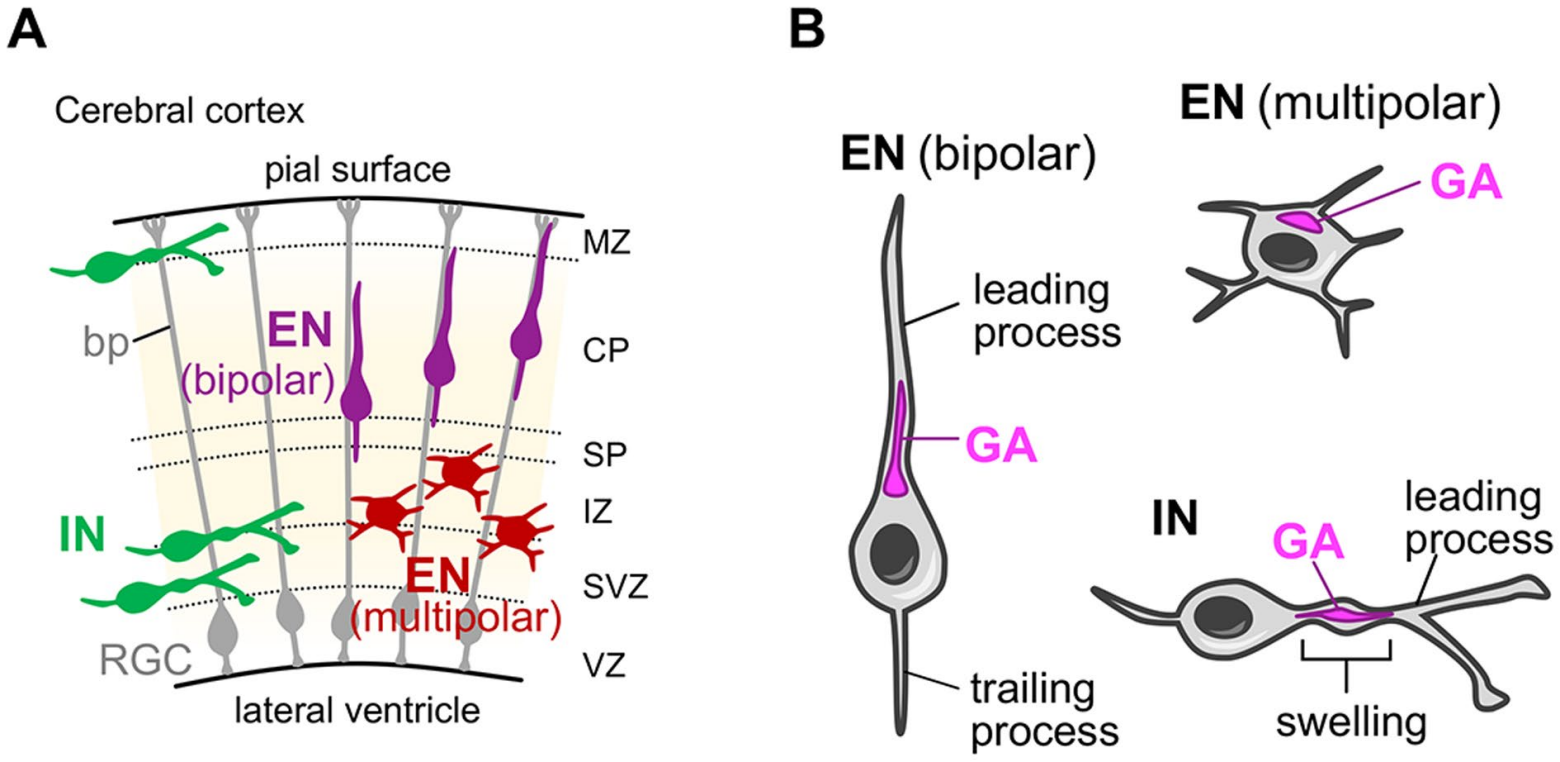
Golgi localization in migrating neurons in the developing cerebral cortex. **(A)** Schematic of the developing mouse cerebral cortex. Radial glial cells (RGCs) with their basal process (bp), excitatory neurons (ENs) in their multipolar and bipolar radial migration phases, and inhibitory interneurons (INs) in their tangential migration phase are shown. CP, cortical plate; IZ, intermediate zone; MZ, marginal zone; SP, subplate; SVZ, subventricular zone; VZ, ventricular zone. **(B)** The localization of the Golgi apparatus in migrating ENs and INs. In migrating ENs during the multipolar phase, the Golgi apparatus (GA) is positioned at the apical side of the nuclei and shows a compact shape. During the bipolar phase, the Golgi in ENs keeps their apical positioning, but shows rod-like shape extending into the leading process. Note that the term “apical” for describing the Golgi position is adopted from the apical-basal axis of the dendrite orientation of mature cortical excitatory neurons. In migrating INs, the Golgi is positioned in the characteristic swelling in the leading process.

After differentiating from neuronal progenitors, the neurons migrate toward the distant region from their birth place. The radial migration of the glutamatergic excitatory neurons in the mammalian cerebral cortex is one of the best studied models for such neuronal migration (Marín et al., 2010; Evsyukova et al., 2013). Cortical excitatory neurons are generated from progenitor cells located at the ventricular side of the cerebral wall in the embryonic dorsal telencephalon and then migrate radially toward the surface of the cortex to form the six-layered cerebral cortex (Figure 3A) (Marín et al., 2010; Evsyukova et al., 2013). During their journey, neurons change their morphology from multipolar to bipolar shape with leading and trailing processes, which eventually develop into dendrites and the axon, respectively (Figure 3B) (Marín et al., 2010; Valiente and Marín, 2010; Evsyukova et al., 2013). The bipolar migration mode (or locomotion mode) relies on the basal processes of radial glial cells, which span the entire cerebral wall and serve as the scaffold for radial neuronal migration (Figure 3A). *In situ* visualization of the Golgi apparatus reveals that, during the multipolar migration stage, the compact-shaped Golgi is positioned at the apical side of the nuclei (Figure 3B) (Jossin and Cooper, 2011) (In this review, the term “apical” for describing the Golgi position is adopted from the apical-basal axis of the dendrite orientation of mature cortical excitatory neurons). During the bipolar migration stage, the Golgi is still positioned at the apical side of the nuclei, but shows rod-like shape extending into the leading process (Figure 3B) (Nichols and Olson, 2010; Franco et al., 2011). These neurons start to elaborate their dendrites to be incorporated into the neocortical circuits after completing radial migration and settling down at their destination (their target cortical layer). Pyramidal neurons, which have a prominent apical dendrite, usually retain the apical Golgi positioning until the postnatal stage (Miao et al., 2013; Nakagawa and Iwasato, 2023). Whereas, stellate neurons exhibit a neuronal activity-dependent change in Golgi distribution (Nakagawa and Iwasato, 2023), which will be described in detail later in section 4.1.

The gamma-aminobutyric acidergic (GABAergic) inhibitory interneurons also show characteristic migration pattern before reaching their final destination. Cortical interneurons are born from progenitors at the ventricular zone of the ganglionic eminence in the subpallium (Marín et al., 2010; Evsyukova et al., 2013; Guo and Anton, 2014). Subsequently, they undergo a tangential, long-distance migration toward the dorsal telencephalon (Figure 3A). They eventually reach their intended regions in the cortical plate after a brief radial migration (Guo and Anton, 2014; Wamsley and Fishell, 2017). During tangential migration, interneurons exhibit bipolar morphology and their leading process undergoes continuous morphological changes, with extensive bifurcations searching for molecular cues (Figure 3A) (Valiente and Marín, 2010; Guo and Anton, 2014). An ultrastructural study has shown that the Golgi apparatus, together with the centrosome, is positioned in the characteristic swelling in the leading process of migrating interneurons (Figure 3B) (Bellion et al., 2005). Furthermore, live-imaging analyses revealed that when interneurons bifurcate the leading process to change their direction, the Golgi apparatus translocates into one of the branches, in a manner preceding the movement of the nucleus into that branch (Yanagida et al., 2012).

Taken together, in both migrating cortical excitatory and inhibitory neurons, the Golgi is positioned at the base of or inside the leading processes. Given that the leading process senses the extracellular guidance cues during migration, the Golgi may fuel the sensing activity by transporting the necessary materials to the cellular antennae.

How is the strategic Golgi positioning regulated in migrating neurons? Studies on the radial migration in the cerebral cortex have revealed several molecular players regulating the Golgi localization in neurons. One well studied upstream regulator is Reelin, a soluble protein secreted from Cajal–Retzius cells in the developing brain (D’Arcangelo et al., 1995; Ogawa et al., 1995; Rice and Curran, 2001). Reelin binds to the neuronal very low density lipoprotein receptor (VLDLR) and apolipoprotein receptor 2 (ApoER2) and activates intracellular adaptor disabled homolog 1 (Dab1) to regulate migration, laminar organization, and morphological maturation of neurons (Howell et al., 1999; D’Arcangelo et al., 1999; Hiesberger et al., 1999). Genetic ablation of Reelin or inhibition of the downstream effector Rap1 causes abnormal Golgi positioning and morphology in radially migrating cortical neurons, which is accompanied by defects in neuronal orientation, migration and layer formation (Matsuki et al., 2010; Nichols and Olson, 2010; Jossin and Cooper, 2011; O’Dell et al., 2012). However, it remains unclear how Reelin signal modulates Golgi positioning and morphology in migrating neurons. Additionally, Bicaudal-D2, an adaptor protein that activates the microtubule-based motor dynein, is necessary for the elongation of Golgi into the leading process (Will et al., 2019). Thus, the minus-end directed motor activity on the microtubule fiber is involved in proper Golgi positioning in migrating neurons. Physical changes in the extracellular environment are also indirectly involved in the regulation of Golgi positioning in migrating neurons. For instance, in polymicrogyria-associated mutant mice, the basal processes of radial glial cells detach from the pial basement membrane and lose their scaffolding function. In this situation, migrating neurons are not able to maintain their bipolar morphology; moreover, the Golgi apparatus shows compact shape at the perinuclear region instead of extending into the leading process (Nakagawa et al., 2015).

To understand the precise role of the Golgi apparatus in neuronal migration, it is necessary to directly disrupt the Golgi apparatus structure/function, instead of manipulating upstream signaling pathway that can influence irrelevant downstream effectors causing off-target effects. There are a few existing studies which directly disrupt the Golgi apparatus structure/function. For example, Matsuki et al. has shown that knockdown of GM130, a Golgi matrix protein necessary for the Golgi ribbon (laterally connected Golgi stacks) formation (Puthenveedu et al., 2006), disrupts radial migration of cortical excitatory neurons (Matsuki et al., 2013). The membrane trafficking function of the Golgi apparatus is also essential for appropriate neuronal migration. Li et al. showed that Sar1b, a small GTPase involved in the anterograde transport from the ER to the Golgi, is required for multipolar-to-bipolar transition of the migration mode in neurons (Li et al., 2020). Additionally, Hara et al. have demonstrated that another small GTPase ADP ribosylation factor 4 (Arf4) localizes at the Golgi apparatus, as well as TGN and recycling endosomes in the migrating neurons (Hara et al., 2023). Inhibiting Arf4 disturbs radial migration of cortical neurons by reducing the surface expression of N-cadherin (Hara et al., 2023), which is a key adhesion molecule in radial migration (Kawauchi et al., 2010; Jossin and Cooper, 2011). Altogether, these findings clearly align with the idea that the proper positioning and membrane trafficking function of the Golgi apparatus control neuronal migration in the developing brain.

## 3 Neuronal Golgi in dendrite specification and elaboration

### 3.1 Specification of primary dendrite

**Figure 4:**
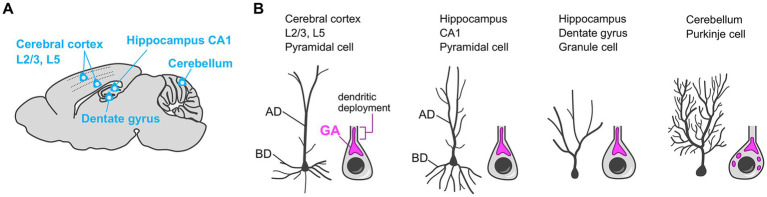
Golgi positioning in diverse types of neurons during the primary dendrite specification. **(A)** Schematic of the sagittal section of the mouse brain. The Golgi localization in the neurons in indicated brain regions are described in **(B)**. **(B)** The typical dendritic morphology (left) and the reported localization of the somatic Golgi apparatus (GA) (right) in cerebral cortex layer 2/3 (L2/3) and L5 pyramidal cells, hippocampal CA1 pyramidal cells, adult-born hippocampal dentate gyrus granule cells, and cerebellar Purkinje cells. These neurons typically have a single primary dendrite and the Golgi apparatus preferentially localizes at the base of the primary dendrite. The Golgi apparatus often elongates into the proximal part of the primary dendrite (dendritic Golgi deployment). AD, apical dendrite; BD, basal dendrite.

Most neurons show developmentally programmed, cell type-specific, and stereotyped dendrite morphology. For example, the pyramidal neuron in the cerebral cortex has a single long and thick apical dendrite, and multiple relatively thin and short basal dendrites. The role of the Golgi apparatus in sculpting such characteristic dendritic architecture, especially in the specification of the primary dendrite (e.g., the apical dendrite of pyramidal neurons), is well studied. In neurons that have a single primary dendrite—such as pyramidal neurons in the hippocampus CA1 and cerebral cortex Layer (L)2/3 and L5 (Horton et al., 2005; Matsuki et al., 2010; Miao et al., 2013), adult-born granule cells in the hippocampal dentate gyrus (Huang et al., 2014; Rao et al., 2018), and Purkinje cells in the cerebellum (Liu et al., 2017; Tanabe et al., 2010)—the Golgi apparatus preferentially localizes at the base of the primary dendrite (Figure 4). Furthermore, the Golgi often elongates into the proximal part of the primary dendrite, which is called the dendritic deployment of the Golgi apparatus (Figure 4). An intriguing question, here, is whether the Golgi is recruited to the established primary dendrite or the Golgi position determines the primary dendrite-initiation site. To address this question, using *in vitro* cultured neurons, researchers performed the simultaneous live-imaging of the neuronal morphology and Golgi distribution (Wu et al., 2015), or they analyze the *in vitro* or *in vivo* spatial relationship between the dendrites and Golgi at different sequential developmental timepoints (Horton et al., 2005; Tanabe et al., 2010). Such studies suggest that the Golgi positioning precedes the primary dendrite specification (Horton et al., 2005; Tanabe et al., 2010; Wu et al., 2015). Therefore, during the primary dendrite specification, it is likely that the Golgi apparatus first translocates to the specific intracellular subdomain in neurons, and then the dendrite initiated from that domain is exclusively elaborated to become the primary dendrite. In agreement with this story, when the Golgi is experimentally dispersed in the soma and thus loses its specific localization, the neuron no longer specifies single neurite as primary dendrite, and instead, shows multiple dendrites with similar length and complexity (Horton et al., 2005; Wu et al., 2015).

How are the polarized Golgi distribution and dendritic Golgi deployment regulated? The Reelin signaling pathway—mentioned earlier, which regulates the Golgi positioning in migrating neurons—importantly regulates the Golgi polarization during primary dendrite specification. In the hippocampal CA1 neuron and cortical pyramidal neurons, the absence of Reelin signaling impairs the Golgi polarization and elongation into the apical dendrite (Matsuki et al., 2010; Franco et al., 2011; Meseke et al., 2013). Reelin binding to neuronal receptors VLDLR and ApoER2 eventually leads to an activation of αPIX, a Rac1/Cdc42-specific guanine nucleotide-exchange factor (GEF). αPIX in turn activates small GTPases Cdc42 and Rac1, both of which regulate the dendritic Golgi deployment (Meseke et al., 2013).

Studies using different model systems reveal several more molecular regulators of Golgi polarization occurring during dendrite specification. In hippocampal pyramidal neurons, the liver kinase B1 (LKB1)-serine/threonine-protein kinase 25 (STK25)-GM130 axis is required for the Golgi deployment into the primary dendrite (Matsuki et al., 2010). This pathway works antagonistically with Reelin signaling. In this situation, the scaffolding function of STK25 (but not its kinase activity) is importantly involved (Matsuki et al., 2010). Moreover, cytoskeletal regulators—including doublecortin, kinesin, RhoA, LIM domain kinase 1 (LIMK1), and coffilin—also participate in the dendritic Golgi deployment (Rosso et al., 2004; Quassollo et al., 2015; Li et al., 2021).

The adult-born granule cell in hippocampal dentate gyrus is also used as a model system to study the primary dendrite specification. The immature adult-born granule cell initially has multiple short dendrites. During maturation, one of these short dendrites is elaborated to form the primary dendrite directed toward the molecular layer; while the other short dendrites are eliminated during maturation (Rao et al., 2018). LKB1 is required for the Golgi deployment into the primary dendrite and for the proper orientation of the primary dendrite toward the molecular layer (Huang et al., 2014). STK25-STE20-related pseudokinase (STRAD) complex is also required for Golgi polarization into the primary dendrite (Rao et al., 2018). Knockdown of either STK25 or STRAD causes abnormal dendrite morphology, it especially causes defects in dendrite elimination (Rao et al., 2018).

Purkinje cells in the cerebellum show a prominent primary dendrite with extensively branched arbors, and thus used as a model to examine dendrite specification mechanisms. During development, Purkinje cells initially have multiple short dendrites, and then retract all but one, which is eventually elaborated to be the primary dendrite (Tanabe et al., 2010; Takeo and Yuzaki, 2021). Tanabe et al. 2010 have shown that the Golgi apparatus is localized at the base of the primary dendrite in zebrafish cerebellar Purkinje cells. They showed that Golgi polarization, which is regulated by atypical protein kinase C (aPKC) (a Par complex component), precedes the specification of the primary dendrite (Tanabe et al., 2010). Liu et al. (2017) have reported that mouse cerebellar Purkinje cells also show apical Golgi localization including deployment into the proximal part of the primary dendrite. This apical Golgi polarity is perturbed in GM130 knockout mice, which is accompanied by severe dendritic atrophy in Purkinje cells, as well as Purkinje cell loss and ataxia (Liu et al., 2017).

Collectively, although some are shared, diverse molecular pathways are identified from different types of neurons. Integrative and comparative analyses of results obtained using different model systems will help elucidate the entire molecular landscape, including shared and cell type-specific pathways, which regulates Golgi polarization occurring during dendrite specification. It is worth noting that the primary dendrite specification and the axon specification (described in section 2.1) have some degree of interdependence with shared regulatory mechanisms. For example, augmentation of Stk25 function or lack of Reelin signaling leads to impaired dendritic Golgi extension, as well as multiple axon induction in hippocampal neurons (Matsuki et al., 2010).

### 3.2 Dendritic arbor elaboration

After the overall dendritic pattern is determined, the neurons acquire the mature and functional dendritic architecture by further dendritic arbor elaboration (elongation, branching, and spine formation). The Golgi apparatus supports such dendritic arbor elaboration by directional vesicle transport. Indeed, live-imaging of the post-Golgi carriers in cultured neurons reveals that fluorescently-labeled cargo proteins exiting from the Golgi are transported into the dendrites (Horton et al., 2005; Al-Bassam et al., 2012). Interestingly, in the neurons that exhibit primary dendritic Golgi polarization, the post-Golgi carriers preferentially move toward the primary dendrite (Horton et al., 2005). In contrast, in neurons that do not show apparent distribution bias of the Golgi apparatus, the post-Golgi carriers seem to evenly enter every dendrite (Al-Bassam et al., 2012). These observations imply that, by positioning to the proper subcellular domain, the Golgi apparatus efficiently sorts the limited resources in the cell to specific dendrite(s) which needs to grow more extensively than the others at the particular developmental stage. The dendrites with the Golgi deployment are indeed longer and more complex than those lacking Golgi (Horton et al., 2005).

The requirement of the Golgi-mediated secretory trafficking for the dendrite growth has been directly tested by pharmacologically or genetically blocking the secretory pathway. In developing cultured hippocampal neurons, BFA treatment results in a severely defective dendritic outgrowth (Horton et al., 2005). Genetic inactivation of the proteins which are involved in the anterograde cargo transport, such as Arf1, PKD, and Sar1, causes a significant reduction in dendrite length (Horton et al., 2005; Ye et al., 2007). Similar experimental strategies were applied to mature neurons to assess the involvement of secretory system on the maintenance of the dendrites. In mature neurons *in vitro*, BFA treatment and PKD inhibition significantly reduces the dendrite length (Horton et al., 2005; Czöndör et al., 2009). These studies clearly illustrate the reliance of both dendrite formation and maintenance on the Golgi-mediated secretory trafficking.

**Figure 5:**
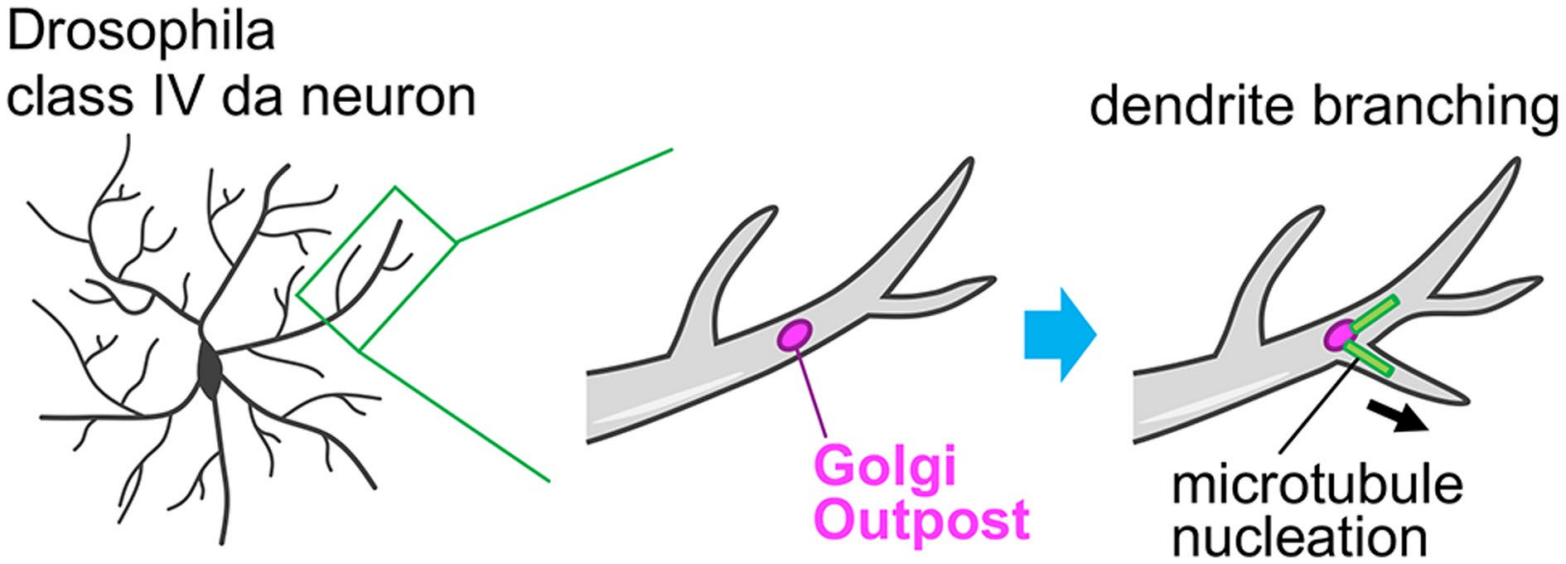
Role of the Golgi outpost in the dendrite branch formation. The class IV dendritic arborization (da) neurons in *Drosophila melanogaster* typically have Golgi outposts throughout their dendritic structures including proximal and distal parts. By acting as a non-centrosomal microtubule nucleation site, these Golgi outposts facilitate the dendrite branching and therefore the complex dendritic arborization of da neurons.

In dendrites, there are small and discrete Golgi units, which are separated from the somatic Golgi apparatus. These units are called Golgi outposts and act as local stations in dendrites in the neuronal secretory pathway (Figures 2, 5) (Kennedy and Hanus, 2019; Valenzuela and Perez, 2015). In mammalian neurons, the Golgi outposts are spatially restricted to the proximal part of the primary dendrite (Figure 2) (Horton et al., 2005; Quassollo et al., 2015; Bowen et al., 2017). These Golgi outposts are generated by fission events at the tip of the tubular structure of the somatic Golgi that is elongating into the primary dendrite, and then they are transported distally (Quassollo et al., 2015). Golgi outpost generation is regulated by the RhoA-Rock signaling pathway, together with LIMK1, PKD1, slingshot, cofilin, and dynamin (Quassollo et al., 2015). In contrast to mammalian neurons, dendritic arborization (da) neurons in *Drosophila melanogaster* show abundant Golgi outposts distributed throughout the entire dendritic arbor length from proximal to distal dendrites (Figure 5) (Ye et al., 2007). Taking advantage of this feature, the function of the Golgi outposts has been primarily examined using *Drosophila* da neurons. It has been found that the Golgi outposts are preferentially localized at the dendritic branch point in class IV da neurons. Furthermore, the dynamics of the Golgi outposts correlate with the dendritic branching behaviors. That is, the *de novo* generation/distal movement or disappearance/proximal movement of the Golgi outposts is followed by dendrite extension or retraction, respectively (Ye et al., 2007). The distribution and dynamics of the Golgi outposts within dendrites are coordinated by the balanced action of dynein and kinesin motors (Ye et al., 2007; Lin et al., 2015; Kelliher et al., 2018). The distal movement is regulated by the dynein system, wherein the Golgi outposts are tethered on dynein/dynactin complex by Lava lamp and this interaction is antagonized by Leucine-rich repeat kinase (Lrrk) proteins (Ye et al., 2007; Lin et al., 2015). Whereas, the proximal movement is directed by kinesin-1, which is normally suppressed by autoinhibition of kinesin-1 to prevent mis-localization of the Golgi outpost to the axon (Kelliher et al., 2018). Laser-mediated ablation of the Golgi outposts leads to reductions of both extension and retraction of dendritic branches. This demonstrates the importance of the Golgi outposts in dendritic branching dynamics and arborization (Ye et al., 2007). Moreover, cargo proteins are indeed released from the dendritic Golgi outposts (Horton et al., 2005), suggesting that the secretory function of the Golgi outposts participates in the dendritic branching. The microtubule-nucleating function is another important aspect of the Golgi outposts. The Golgi apparatus acts as a non-centrosomal microtubule organization center (Chabin-Brion et al., 2001; Efimov et al., 2007; Wu and Akhmanova, 2017). Ori-McKenney et al. (2012) showed that Golgi outposts can directly nucleate microtubules in dendrites, which contributes to the extension and stabilization of dendritic branch (Figure 5). Therefore, the dendritic Golgi outposts serve as the remote machinery that fine-tunes dendritic architecture and complexity.

## 4 Neuronal Golgi in activity-dependent plasticity

### 4.1 Activity-dependent dendrite patterning

The rough framework of the neural circuit, including initial projection pattern of neuronal axons and dendrites, are formed during embryonic stages, which largely depends on the genetic programs (e.g., gradient of diffusible morphogens and area/cell type-specific transcription factor expression) (Greig et al., 2013; Cadwell et al., 2019). Subsequently, during the postnatal stage, neural circuits undergo extensive refinement, which is achieved by morphological and functional remodeling of neurons. This postnatal neuron remodeling is critically dependent on the neuronal activity evoked either spontaneously or by extrinsic stimuli (Goodman and Shatz, 1993; Katz and Shatz, 1996; Wong and Ghosh, 2002). Remodeling of dendrites is one of the key cellular mechanisms for postnatal circuit refinement, which enables reinforcement and elimination of specific and non-specific synaptic connections, respectively, thereby optimizing receptive fields of the neuron (Wong and Ghosh, 2002; Nakagawa and Iwasato, 2024). The rearrangement of the dendrite structure is thought to be a general strategy for sculpting mature connectivity in the nervous system as it is conserved across diverse neuronal types, brain regions, and species (Cline, 2001; Wong and Ghosh, 2002; Emoto, 2011).

**Figure 6:**
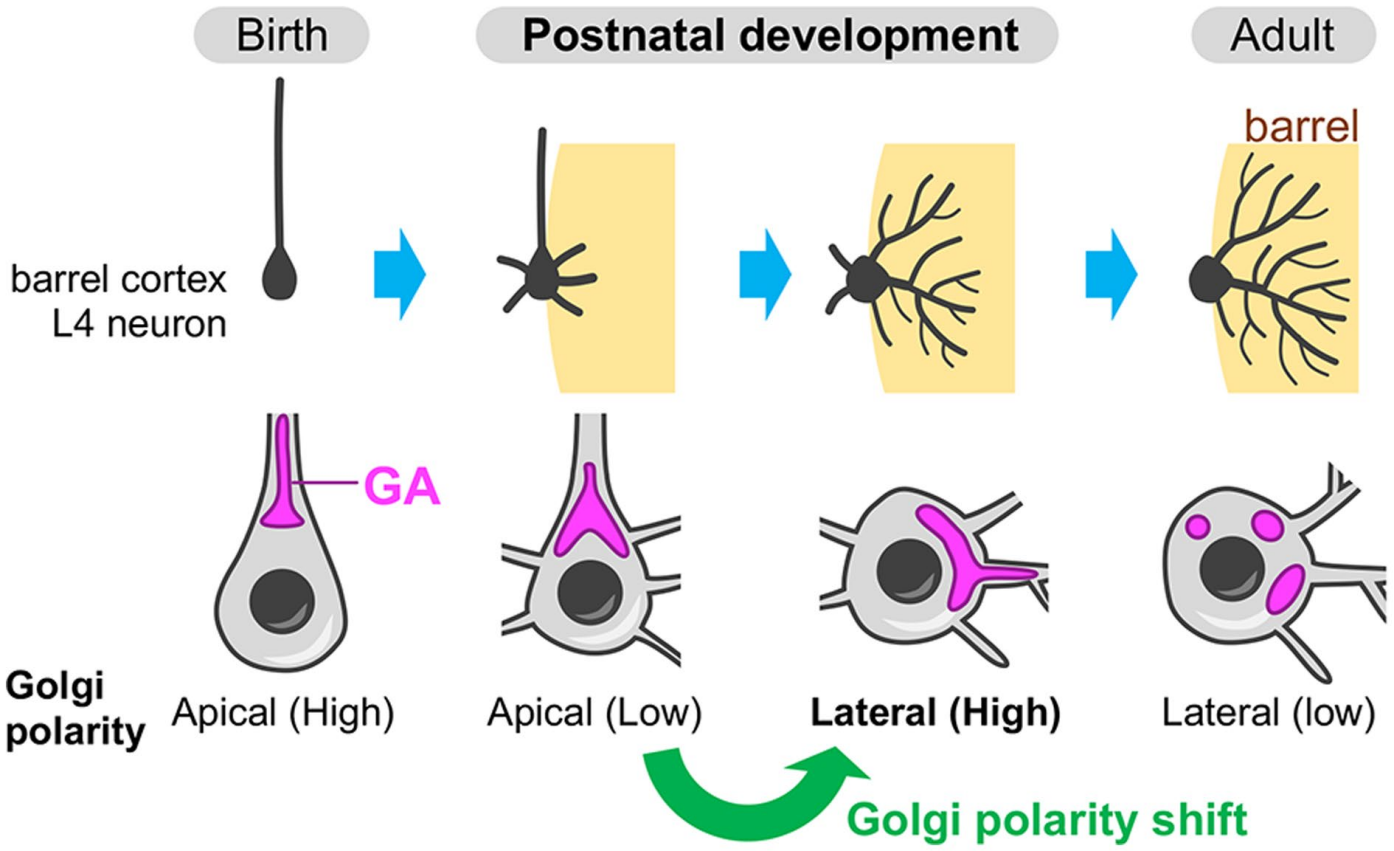
Neuronal activity-dependent shift of the Golgi polarity during postnatal circuit reorganization. In the barrel cortex layer 4 (L4), spiny stellate neurons (a major subtype of the excitatory neurons in the barrel cortex L4) initially have the Golgi apparatus (GA) in the apical side of the soma (apical Golgi polarity) (birth). During postnatal development, these neurons break down the initial apical polarity and shift it to the lateral direction, which is oriented toward a single barrel (the region where the target thalamocortical axon terminals are clustering). The Golgi is often deployed into the dendrite(s) that is extending toward the barrel. Finally, after the completion of circuit reorganization, spiny stellate neurons decrease the lateral Golgi polarity (adult).

Recently, the Golgi apparatus, especially its developmental dynamics, has been shown to play an instructive role in the postnatal dendrite rearrangement (Nakagawa and Iwasato, 2023). The evidence is obtained from the study using mouse barrel cortex—a major part of the primary somatosensory cortex, which processes whisker-derived tactile information. The L4 spiny stellate neurons in the barrel cortex undergo extensive refinement of their basal dendrites during the first postnatal week to acquire an asymmetric dendritic projection toward a single barrel, which specifically processes sensory information from corresponding single whisker (reviewed in Iwasato, 2020; Nakagawa and Iwasato, 2024). In L4 spiny stellate neurons, the Golgi apparatus initially resides in the apical domain until around postnatal day 3 (P3), then translocates to the lateral domain, especially toward a single barrel by P5 (Figure 6) (Nakagawa and Iwasato, 2023). Thus, L4 spiny stellate neurons show an apical-to-lateral shift of the Golgi polarity, and the resulting lateral Golgi polarity diminishes along with the completion of dendritic refinement, by the third postnatal week (Figure 6). This lateral Golgi polarization is dependent on the N-methyl-D-aspartate-type glutamate receptor (NMDAR) activity induced by presynaptic thalamocortical inputs. In the barrel cortex L4, there is a patchwork-type spontaneous neuronal activity during the first postnatal week, wherein the L4 excitatory neurons within the same barrel fire synchronously (Mizuno et al., 2018; Nakazawa et al., 2020). During this period, the presynaptic thalamocortical axon terminals that belong to the same barrel also fire synchronously (Mizuno et al., 2018). Thus, in L4 spiny stellate neurons, the basal dendrites formed on the barrel-side seem to receive strong synchronous inputs, which likely create NMDAR signaling gradients and thereby generate lateral Golgi polarity. The laterally polarized Golgi is necessary for the L4 spiny stellate neurons to acquire asymmetric basal dendrite projection toward single barrels, as well as the specific response to principal whisker stimulation (Nakagawa and Iwasato, 2023). The secretory function of the Golgi is likely involved in this dendrite asymmetry formation. This is evidenced by the fact that the dendritic trees that contain the Golgi at their base (or inside) are more elaborated than those without Golgi (Nakagawa and Iwasato, 2023). Neuronal activity-mediated Golgi re-distribution illustrates that the Golgi apparatus is not static but plastic even in postnatal stages. Postnatal Golgi plasticity can serve as a cellular basis in adaptive remodeling of brain circuitry and functions.

### 4.2 Activity-induced Golgi fragmentation and Golgi satellite formation

Several lines of evidence indicate that the Golgi apparatus structure and function in neurons are modulated by non-physiological (experimental or disease-associated) hyperexcitation. For example, hippocampal and cortical neurons in culture show fragmentation of the Golgi apparatus when treated with elevated potassium ion concentration, which recapitulates hyperexcitable condition (Thayer et al., 2013; Mohamed et al., 2017). The Golgi fragmentation is also observed upon experimental activation of neurons, such as treatment with GABA_A_ receptor antagonist bicuculline or removal of NMDAR antagonist APV after its prolonged exposure (Thayer et al., 2013). This experimental Golgi fragmentation is reversible, and dependent on Ca^2+^/calmodulin-dependent protein kinase (CaMK) II/IV (Thayer et al., 2013). High potassium treatment activates cyclin-dependent kinase 5 (Cdk5) (Mohamed et al., 2017), which may be mediated by Ca^2+^ influx-activated calpain (Lee et al., 2000). Activated Cdk5 phosphorylates GM130 (Sun et al., 2008; Mohamed et al., 2017). GM130 phosphorylation causes the Golgi fragmentation during mitosis (Lowe et al., 1998). Thus, neuronal hyperactivation and subsequent Ca^2+^ influx leads to activation of CaMKII/IV and Cdk5 pathways, which act in concert or in parallel to cause the Golgi fragmentation in neurons.

The results of the above studies conducted using a cellular model provide implications into some disease-associated Golgi abnormalities. For instance, epilepsy is a complex neurological disorder accompanied by recurrent seizures due to hypersynchronous activation of neurons (Stafstrom and Carmant, 2015). The Golgi apparatus is fragmented and scattered throughout the soma of the neocortical pyramidal neurons in human patients with intractable epilepsy (Skupien-Jaroszek et al., 2023). Experimental induction of epileptic seizures in rats by kainic acid injection recapitulates the Golgi fragmentation in hippocampal pyramidal and dentate gyrus granule neurons (Skupien-Jaroszek et al., 2023). Moreover, *in vitro* cultured hippocampal and cortical neurons also show the Golgi fragmentation upon kainic acid treatment (Kaneko et al., 2016; Skupien-Jaroszek et al., 2023). Kainic acid-induced Golgi dispersion is largely rescued by either treating with an antiepileptic drug or chelating intracellular Ca^2+^ (Kaneko et al., 2016; Skupien-Jaroszek et al., 2023). Kainic acid treatment results in a reduction of the protein amount of GM130 (Kaneko et al., 2016). Thus, Ca^2+^ influx followed by neuronal hyperexcitation somehow reduces the GM130 protein level, which can cause Golgi fragmentation in the brains of patients with epilepsy.

Besides epilepsy, the Golgi fragmentation in neurons is observed in many neurodegenerative disorders, including Alzheimer’s disease, Parkinson’s disease, and amyotrophic lateral sclerosis (ALS) (Stieber et al., 1996; Fujita and Okamoto, 2005; Fujita et al., 2006; Tomás et al., 2021; Caracci et al., 2019). Considering that the hyperexcitability of neurons is a common hallmark among these neurodegenerative disorders (DeLong and Wichmann, 2007; Geevasinga et al., 2016; Targa Dias Anastacio et al., 2022), the above-described, abnormal hyperactivity-induced mechanisms of the Golgi fragmentation may be shared in deteriorated neurons in these disorders.

In contrast to the well-documented morphological defects of the Golgi in neurological disorders, our understanding about how these Golgi defects relate to disease pathogenesis is still limited. So far, we know that Golgi fragmentation perturbs the distribution of Golgi-resident enzymes and hence the glycosylation during secretory pathway (Puthenveedu et al., 2006). Moreover, in regard to the Alzheimer’s disease, the Golgi fragmentation enhances amyloid beta production and tau secretion, which may accelerate the disease progression (Joshi et al., 2014; Mohamed et al., 2017). Further studies are required to connect the hyperexcitability-induced or pathological Golgi alterations to the cellular pathogenesis of neurological disorders.

Another kind of Golgi alteration triggered by neuronal activity is the formation of the Golgi satellites in dendrites. The Golgi satellites are typically smaller than the Golgi outposts and widely distributed throughout the dendrites of mammalian neurons, including secondary and tertiary arbors, with less preference to the dendritic branchpoint (Mikhaylova et al., 2016). The Golgi satellites contain glycosylation enzymes (e.g., sialyltransferase) but lack conventional marker proteins for somatic Golgi and Golgi outposts, such as GM130 (Mikhaylova et al., 2016). In cultured rat cortical neurons, excitatory stimulation enhances the formation of Golgi satellites (Govind et al., 2021). Newly synthesized cargo proteins pass through the dendritic Golgi satellite before reaching the plasma membrane (Mikhaylova et al., 2016; Govind et al., 2021). The Golgi satellites also seem to be engaged in the recycling of neurotransmitter receptors and conversion of the surface glycoproteome to more complex form (Mikhaylova et al., 2016; Govind et al., 2021). Thus, the Golgi satellites serve as the micro-secretory system in dendrites, which enables local activity-dependent remodeling of the post-translational modification and combination of the neuronal surface proteins in response to the local demands.

## 5 Future perspective and conclusion

In this review, I have summarized the characteristic localization of the Golgi apparatus and Golgi-related structures (Golgi outposts and satellites) in neurons during each developmental milestone, including the axon specification, migration, primary dendrite specification, dendrite arbor elaboration, postnatal dendrite remodeling, plastic response to excitatory stimuli, and alterations in neurological disorders. The molecular mechanisms regulating the Golgi distribution and functional roles of the Golgi apparatus in neurons are also discussed at each developmental stage.

The research on the Golgi apparatus as an organelle has a long history, starting from its discovery by Camillo Golgi in 1898, and the roles played by the neuronal Golgi in the initial neural circuit formation are extensively studied. Especially, the distribution and function of the Golgi in earlier (mostly embryonic) developmental events in neurons, such as initial polarization, migration, and dendrite specification/elaboration, are relatively well characterized. In contrast, recent discoveries reveal that the Golgi apparatus is critically involved in activity-dependent remodeling of neuronal morphology/function during postnatal neural circuit reorganization, in addition to its roles in the genetically programmed early developmental events. The plastic nature of the Golgi apparatus in physiological condition is just being unraveled. For example, the Golgi polarity shift that regulates dendrite remodeling has only been described in the mouse barrel cortex L4 spiny stellate neurons at this moment (Nakagawa and Iwasato, 2023). Further efforts will be needed to examine whether this Golgi polarity shift and its associated roles are conserved across different types of neurons in different brain areas or across species. Moreover, the activity-induced Golgi satellite formation that remodels dendritic surface glycoproteome is currently only shown in *in vitro* cultured neurons (Govind et al., 2021). It will be intriguing to examine whether and how this system is implemented to support adaptive changes in neurons *in vivo*. Accumulating molecular insights on plastic changes of the Golgi apparatus will bring clues from a different angle which will deepen our understanding of the causal relationships between the Golgi abnormalities and cellular pathogenesis of devastating neurological disorders.

In future, the study on the Golgi apparatus in neural circuit formation and function needs to unravel the following points. First, it is important to reveal the molecular nature of the secretory function of the Golgi apparatus. We now know that the secretory vesicles derived from the Golgi play critical roles in various developmental events, such as specification and elaboration of the axon and dendrites. However, are there any key molecules in the secretory vesicles, which play major role in a particular developmental event? We also do not know if these key molecules are shared/differ between developmental events. So far it had been difficult for us to explore these questions because of the lack of the methodology to comprehensively identify the molecules transported from the Golgi apparatus in a defined cell type *in vivo* in a restricted developmental time window. Recent innovations in the strategy to target genetically defined neuronal types and in the single-cell multi-omics approaches (including proteomics and lipidomics) may allow us to overcome the technical limitation. Knowledge of these key issues will shift our understanding of the role of the Golgi from knowing it as a transport machinery to knowing it as a supplier of tailored molecules, specific to individual developmental events.

Second, it is important to reveal the role of the Golgi in the morphological maturation and function of the GABAergic inhibitory neurons. Currently, we know that the Golgi apparatus contributes to the morphological maturation of cortical and hippocampal excitatory neurons (Horton et al., 2005; Matsuki et al., 2010; Nakagawa and Iwasato, 2023). However, the evidence about the role of the Golgi in the morphological maturation and function of the GABAergic inhibitory neurons in these brain areas is still lacking. An earlier work has suggested that there is no obvious Golgi polarization in developing GABAergic interneurons *in vitro* (Horton et al., 2005). Recently, it has been revealed that GABAergic interneurons in cerebral cortex and hippocampus can be categorized into diverse subtypes, which show distinct morphology, electrophysiological feature, and gene expression profile (Tremblay et al., 2016; Wamsley and Fishell, 2017; Gouwens et al., 2020; Tzilivaki et al., 2023). Thus, analyzing the Golgi apparatus distribution and its developmental changes in morphologically and/or transcriptomically defined inhibitory neuron subtypes may identify potential contribution of the Golgi to inhibitory circuit formation and refinement.

Third, it will be interesting to see the positioning and functioning of the Golgi apparatus in other brain cell types, such as astrocyte, oligodendrocyte, and microglia. The normal function of our brain is influenced by these nonneuronal cells, in addition to neurons. Thus, understanding the contribution of nonneuronal cells is equally important. Several pioneering electron microscopy and immunohistochemical studies have identified that astrocytes have the Golgi apparatus not only in the cell body but also in peripheral processes (Stieber et al., 1987; Lavi et al., 1994; Kemal et al., 2022). A recent study demonstrate that oligodendrocytes also have the Golgi outposts in their distal processes, which are marked by tubulin polymerization promoting protein (TPPP), as well as GM130 (Fu et al., 2019). TPPP is essential for microtubule nucleation from the Golgi outposts, and genetic ablation of TPPP in mice results in hypomyelination with poor myelin sheaths and motor coordination deficits (Fu et al., 2019). Therefore, the Golgi apparatus in glial cells seems to play indispensable roles in their morphological development and physiological functions.

In conclusion, the polarized Golgi positioning and functioning enables morphological specification of neurons (and glial cells as well) and generates functional microdomains in their membrane. Therefore, focusing on the role of the Golgi apparatus in neurons, glial cells, and other cell types in the nervous system will offer a unique approach to understand how these different cell types develop and interact with each other to form and remodel the neural circuits.

## References

[ref1] Al-Bassam S. Xu M. Wandless T. J. Arnold D. B. (2012). Differential trafficking of transport vesicles contributes to the localization of dendritic proteins. Cell Rep. 2, 89–100. doi: 10.1016/j.celrep.2012.05.018, PMID: 22840400 PMC3408588

[ref2] Aridor M. Fish K. N. (2009). Selective targeting of ER exit sites supports axon development. Traffic 10, 1669–1684. doi: 10.1111/j.1600-0854.2009.00974.x, PMID: 19761544 PMC2763039

[ref3] Arimura N. Kaibuchi K. (2007). Neuronal polarity: from extracellular signals to intracellular mechanisms. Nat. Rev. Neurosci. 8, 194–205. doi: 10.1038/nrn2056, PMID: 17311006

[ref4] Barnes A. P. Polleux F. (2009). Establishment of axon-dendrite polarity in developing neurons. Annu. Rev. Neurosci. 32, 347–381. doi: 10.1146/annurev.neuro.31.060407.125536, PMID: 19400726 PMC3170863

[ref5] Bellion A. Baudoin J. P. Alvarez C. Bornens M. Métin C. (2005). Nucleokinesis in tangentially migrating neurons comprises two alternating phases: forward migration of the Golgi/centrosome associated with centrosome splitting and myosin contraction at the rear. J. Neurosci. 25, 5691–5699. doi: 10.1523/JNEUROSCI.1030-05.2005, PMID: 15958735 PMC6724882

[ref6] Bisbal M. Conde C. Donoso M. Bollati F. Sesma J. Quiroga S.. (2008). Protein kinase d regulates trafficking of dendritic membrane proteins in developing neurons. J. Neurosci. 28, 9297–9308. doi: 10.1523/JNEUROSCI.1879-08.2008, PMID: 18784310 PMC2648138

[ref7] Boncompain G. Weigel A. V. (2018). Transport and sorting in the Golgi complex: multiple mechanisms sort diverse cargo. Curr. Opin. Cell Biol. 50, 94–101. doi: 10.1016/j.ceb.2018.03.002, PMID: 29567348

[ref8] Bowen A. B. Bourke A. M. Hiester B. G. Hanus C. Kennedy M. J. (2017). Golgi-independent secretory trafficking through recycling endosomes in neuronal dendrites and spines. eLife 6:e27362. doi: 10.7554/eLife.27362, PMID: 28875935 PMC5624785

[ref9] Bradke F. Dotti C. G. (1997). Neuronal polarity: vectorial cytoplasmic flow precedes axon formation. Neuron 19, 1175–1186. doi: 10.1016/S0896-6273(00)80410-9, PMID: 9427242

[ref10] Cadwell C. R. Bhaduri A. Mostajo-Radji M. A. Keefe M. G. Nowakowski T. J. (2019). Development and arealization of the cerebral cortex. Neuron 103, 980–1004. doi: 10.1016/j.neuron.2019.07.009, PMID: 31557462 PMC9245854

[ref11] Caracci M. O. Fuentealba L. M. Marzolo M. P. (2019). Golgi complex dynamics and its implication in prevalent neurological disorders. Front. Cell Dev. Biol. 7:75. doi: 10.3389/fcell.2019.00075 31134199 PMC6514153

[ref12] Chabin-Brion K. Marceiller J. Perez F. Settegrana C. Drechou A. Durand G.. (2001). The Golgi complex is a microtubule-organizing organelle. Mol. Biol. Cell 12, 2047–2060. doi: 10.1091/mbc.12.7.2047 11452002 PMC55652

[ref13] Cline H. T. (2001). Dendritic arbor development and synaptogenesis. Curr. Opin. Neurobiol. 11, 118–126. doi: 10.1016/S0959-4388(00)00182-3, PMID: 11179881

[ref14] Consalez G. G. Goldowitz D. Casoni F. Hawkes R. (2021). Origins, development, and compartmentation of the granule cells of the cerebellum. Front. Neural. Circuits. 14:611841. doi: 10.3389/fncir.2020.611841, PMID: 33519389 PMC7843939

[ref15] Czöndör K. Ellwanger K. Fuchs Y. F. Lutz S. Gulyás M. Mansuy I. M.. (2009). Protein kinase D controls the integrity of Golgi apparatus and the maintenance of dendritic arborization in hippocampal neurons. Mol. Biol. Cell 20, 2108–2120. doi: 10.1091/mbc.e08-09-0957 19211839 PMC2663930

[ref16] D’Arcangelo G. Homayouni R. Keshvara L. Rice D. S. Sheldon M. Curran T. (1999). Reelin is a ligand for lipoprotein receptors. Neuron 24, 471–479. doi: 10.1016/S0896-6273(00)80860-0, PMID: 10571240

[ref17] D’Arcangelo G. Miao G. G. Chen S. C. Soares H. D. Morgan J. I. Curran T. (1995). A protein related to extracellular matrix proteins deleted in the mouse mutant reeler. Nature 374, 719–723. doi: 10.1038/374719a0, PMID: 7715726

[ref18] de Anda F. C. Pollarolo G. Da Silva J. S. Camoletto P. G. Feiguin F. Dotti C. G. (2005). Centrosome localization determines neuronal polarity. Nature 436, 704–708. doi: 10.1038/nature03811, PMID: 16079847

[ref19] DeLong M. R. Wichmann T. (2007). Circuits and circuit disorders of the basal ganglia. Arch. Neurol. 64, 20–24. doi: 10.1001/archneur.64.1.20, PMID: 17210805

[ref20] Deng C. Y. Lei W. L. Xu X. H. Ju X. C. Liu Y. Luo Z. G. (2014). JIP1 mediates anterograde transport of Rab10 cargos during neuronal polarization. J. Neurosci. 34, 1710–1723. doi: 10.1523/JNEUROSCI.4496-13.2014, PMID: 24478353 PMC8186508

[ref21] Dotti C. G. Sullivan C. A. Banker G. A. (1988). The establishment of polarity by hippocampal neurons in culture. J. Neurosci. 8, 1454–1468. doi: 10.1523/JNEUROSCI.08-04-01454.1988, PMID: 3282038 PMC6569279

[ref22] Efimov A. Kharitonov A. Efimova N. Loncarek J. Miller P. M. Andreyeva N.. (2007). Asymmetric CLASP-dependent nucleation of noncentrosomal microtubules at the trans-Golgi network. Dev. Cell 12, 917–930. doi: 10.1016/j.devcel.2007.04.002, PMID: 17543864 PMC2705290

[ref23] Emoto K. (2011). Dendrite remodeling in development and disease. Develop. Growth Differ. 53, 277–286. doi: 10.1111/j.1440-169X.2010.01242.x, PMID: 21492146

[ref24] Evsyukova I. Plestant C. Anton E. S. (2013). Integrative mechanisms of oriented neuronal migration in the developing brain. Annu. Rev. Cell Dev. Biol. 29, 299–353. doi: 10.1146/annurev-cellbio-101512-122400, PMID: 23937349 PMC3930923

[ref25] Ferreira A. Niclas J. Vale R. D. Banker G. Kosik K. S. (1992). Suppression of kinesin expression in cultured hippocampal neurons using antisense oligonucleotides. J. Cell Biol. 117, 595–606. doi: 10.1083/jcb.117.3.595, PMID: 1533397 PMC2289440

[ref26] Franco S. J. Martinez-Garay I. Gil-Sanz C. Harkins-Perry S. R. Müller U. (2011). Reelin regulates cadherin function via Dab1/Rap1 to control neuronal migration and lamination in the neocortex. Neuron 69, 482–497. doi: 10.1016/j.neuron.2011.01.003, PMID: 21315259 PMC3056352

[ref27] Fu M. M. McAlear T. S. Nguyen H. Oses-Prieto J. A. Valenzuela A. Shi R. D.. (2019). The Golgi outpost protein TPPP nucleates microtubules and is critical for myelination. Cell 179, 132–146.e14. doi: 10.1016/j.cell.2019.08.025, PMID: 31522887 PMC7214773

[ref28] Fujita Y. Ohama E. Takatama M. Al-Sarraj S. Okamoto K. (2006). Fragmentation of Golgi apparatus of nigral neurons with alpha-synuclein-positive inclusions in patients with Parkinson's disease. Acta Neuropathol. 112, 261–265. doi: 10.1007/s00401-006-0114-4, PMID: 16855830

[ref29] Fujita Y. Okamoto K. (2005). Golgi apparatus of the motor neurons in patients with amyotrophic lateral sclerosis and in mice models of amyotrophic lateral sclerosis. Neuropathology 25, 388–394. doi: 10.1111/j.1440-1789.2005.00616.x, PMID: 16382790

[ref30] Geevasinga N. Menon P. Özdinler P. H. Kiernan M. C. Vucic S. (2016). Pathophysiological and diagnostic implications of cortical dysfunction in ALS. Nat. Rev. Neurol. 12, 651–661. doi: 10.1038/nrneurol.2016.140, PMID: 27658852

[ref31] Goodman C. S. Shatz C. J. (1993). Developmental mechanisms that generate precise patterns of neuronal connectivity. Cell 72, 77–98. doi: 10.1016/S0092-8674(05)80030-3, PMID: 8428376

[ref32] Gouwens N. W. Sorensen S. A. Baftizadeh F. Budzillo A. Lee B. R. Jarsky T.. (2020). Integrated Morphoelectric and transcriptomic classification of cortical GABAergic cells. Cell 183, 935–953. doi: 10.1016/j.cell.2020.09.057, PMID: 33186530 PMC7781065

[ref33] Govind A. P. Jeyifous O. Russell T. A. Yi Z. Weigel A. V. Ramaprasad A.. (2021). Activity-dependent Golgi satellite formation in dendrites reshapes the neuronal surface glycoproteome. eLife 10:e68910. doi: 10.7554/eLife.68910, PMID: 34545811 PMC8494481

[ref34] Greig L. C. Woodworth M. B. Galazo M. J. Padmanabhan H. Macklis J. D. (2013). Molecular logic of neocortical projection neuron specification, development and diversity. Nat. Rev. Neurosci. 14, 755–769. doi: 10.1038/nrn3586, PMID: 24105342 PMC3876965

[ref35] Guo J. Anton E. S. (2014). Decision making during interneuron migration in the developing cerebral cortex. Trends Cell Biol. 24, 342–351. doi: 10.1016/j.tcb.2013.12.001, PMID: 24388877 PMC4299592

[ref36] Guo Y. Sirkis D. W. Schekman R. (2014). Protein sorting at the trans-Golgi network. Annu. Rev. Cell Dev. Biol. 30, 169–206. doi: 10.1146/annurev-cellbio-100913-013012, PMID: 25150009

[ref37] Hara Y. Katsuyama T. Fukaya M. Sugawara T. Shiroshima T. Sadakata T.. (2023). ADP Ribosylation factor 4 (Arf4) regulates radial migration through N-cadherin trafficking during cerebral cortical development. eNeuro 10:11. doi: 10.1523/ENEURO.0125-23.2023 PMC10630928 37848288

[ref38] Hiesberger T. Trommsdorff M. Howell B. W. Goffinet A. Mumby M. C. Cooper J. A.. (1999). Direct binding of Reelin to VLDL receptor and ApoE receptor 2 induces tyrosine phosphorylation of disabled-1 and modulates tau phosphorylation. Neuron 24, 481–489. doi: 10.1016/S0896-6273(00)80861-2, PMID: 10571241

[ref39] Horton A. C. Racz B. Monson E. E. Lin A. L. Weinberg R. J. Ehlers M. D. (2005). Polarized secretory trafficking directs cargo for asymmetric dendrite growth and morphogenesis. Neuron 48, 757–771. doi: 10.1016/j.neuron.2005.11.005, PMID: 16337914

[ref40] Howell B. W. Herrick T. M. Cooper J. A. (1999). Reelin-induced tyrosine [corrected] phosphorylation of disabled 1 during neuronal positioning. Genes Dev. 13, 643–648. doi: 10.1101/gad.13.6.643, PMID: 10090720 PMC316552

[ref41] Huang W. She L. Chang X. Y. Yang R. R. Wang L. Ji H. B.. (2014). Protein kinase LKB1 regulates polarized dendrite formation of adult hippocampal newborn neurons. Proc. Natl. Acad. Sci. U. S. A. 111, 469–474. doi: 10.1073/pnas.1321454111, PMID: 24367100 PMC3890881

[ref42] Huber L. A. Dupree P. Dotti C. G. (1995). A deficiency of the small GTPase rab8 inhibits membrane traffic in developing neurons. Mol. Cell. Biol. 15, 918–924. doi: 10.1128/MCB.15.2.918, PMID: 7823956 PMC231976

[ref43] Iwasato T. (2020). In vivo imaging of neural circuit formation in the neonatal mouse barrel cortex. Develop. Growth Differ. 62, 476–486. doi: 10.1111/dgd.12693, PMID: 33032363

[ref44] Jareb M. Banker G. (1997). Inhibition of axonal growth by brefeldin a in hippocampal neurons in culture. J. Neurosci. 17, 8955–8963. doi: 10.1523/JNEUROSCI.17-23-08955.1997, PMID: 9364043 PMC6573598

[ref45] Joshi G. Chi Y. Huang Z. Wang Y. (2014). Aβ-induced Golgi fragmentation in Alzheimer's disease enhances Aβ production. Proc. Natl. Acad. Sci. U. S. A. 111, E1230–E1239. doi: 10.1073/pnas.1320192111 24639524 PMC3977293

[ref46] Jossin Y. Cooper J. A. (2011). Reelin, Rap1 and N-cadherin orient the migration of multipolar neurons in the developing neocortex. Nat. Neurosci. 14, 697–703. doi: 10.1038/nn.2816, PMID: 21516100 PMC3102785

[ref47] Kaneko Y. Sullivan R. Dailey T. Vale F. L. Tajiri N. Borlongan C. V. (2016). Kainic acid-induced Golgi complex fragmentation/dispersal shifts the proteolysis of Reelin in primary rat neuronal cells: an in vitro model of early stage epilepsy. Mol. Neurobiol. 53, 1874–1883. doi: 10.1007/s12035-015-9126-1 25790952 PMC4577368

[ref48] Katz L. C. Shatz C. J. (1996). Synaptic activity and the construction of cortical circuits. Science 274, 1133–1138. doi: 10.1126/science.274.5290.1133, PMID: 8895456

[ref49] Kawauchi T. Sekine K. Shikanai M. Chihama K. Tomita K. Kubo K.. (2010). Rab GTPases-dependent endocytic pathways regulate neuronal migration and maturation through N-cadherin trafficking. Neuron 67, 588–602. doi: 10.1016/j.neuron.2010.07.007, PMID: 20797536

[ref50] Kelliher M. T. Yue Y. Ng A. Kamiyama D. Huang B. Verhey K. J.. (2018). Autoinhibition of kinesin-1 is essential to the dendrite-specific localization of Golgi outposts. J. Cell Biol. 217, 2531–2547. doi: 10.1083/jcb.201708096, PMID: 29728423 PMC6028532

[ref51] Kemal S. Richardson H. S. Dyne E. D. Fu M. M. (2022). ER and Golgi trafficking in axons, dendrites, and glial processes. Curr. Opin. Cell Biol. 78:102119. doi: 10.1016/j.ceb.2022.102119, PMID: 35964523 PMC9590103

[ref52] Kennedy M. J. Hanus C. (2019). Architecture and dynamics of the neuronal secretory network. Annu. Rev. Cell Dev. Biol. 35, 543–566. doi: 10.1146/annurev-cellbio-100818-125418, PMID: 31283381 PMC6935261

[ref53] Laurino L. Wang X. X. de la Houssaye B. A. Sosa L. Dupraz S. Cáceres A.. (2005). PI3K activation by IGF-1 is essential for the regulation of membrane expansion at the nerve growth cone. J. Cell Sci. 118, 3653–3662. doi: 10.1242/jcs.02490, PMID: 16046480

[ref54] Lavi E. Wang Q. Stieber A. Gonatas N. K. (1994). Polarity of processes with Golgi apparatus in a subpopulation of type I astrocytes. Brain Res. 647, 273–285. doi: 10.1016/0006-8993(94)91327-7, PMID: 7922504 PMC7111168

[ref55] Lecuit T. Pilot F. (2003). Developmental control of cell morphogenesis: a focus on membrane growth. Nat. Cell Biol. 5, 103–108. doi: 10.1038/ncb0203-103, PMID: 12563275

[ref56] Lee M. S. Kwon Y. T. Li M. Peng J. Friedlander R. M. Tsai L. H. (2000). Neurotoxicity induces cleavage of p35 to p25 by calpain. Nature 405, 360–364. doi: 10.1038/35012636, PMID: 10830966

[ref57] Lefcort F. Bentley D. (1989). Organization of cytoskeletal elements and organelles preceding growth cone emergence from an identified neuron in situ. J. Cell Biol. 108, 1737–1749. doi: 10.1083/jcb.108.5.1737, PMID: 2654140 PMC2115544

[ref58] Li P. Li L. Yu B. Wang X. Wang Q. Lin J.. (2021). Doublecortin facilitates the elongation of the somatic Golgi apparatus into proximal dendrites. Mol. Biol. Cell 32, 422–434. doi: 10.1091/mbc.E19-09-0530 33405953 PMC8098852

[ref59] Li X. Yan M. Guo Z. Yan L. Feng R. Zhu H.. (2020). Inhibition of Sar1b, the gene implicated in chylomicron retention disease, impairs migration and morphogenesis of developing cortical neurons. Neuroscience 449, 228–240. doi: 10.1016/j.neuroscience.2020.09.044, PMID: 33002559

[ref60] Lin C. H. Li H. Lee Y. N. Cheng Y. J. Wu R. M. Chien C. T. (2015). Lrrk regulates the dynamic profile of dendritic Golgi outposts through the golgin lava lamp. J. Cell Biol. 210, 471–483. doi: 10.1083/jcb.201411033, PMID: 26216903 PMC4523617

[ref61] Liu C. Mei M. Li Q. Roboti P. Pang Q. Ying Z.. (2017). Loss of the golgin GM130 causes Golgi disruption, Purkinje neuron loss, and ataxia in mice. Proc. Natl. Acad. Sci. U. S. A. 114, 346–351. doi: 10.1073/pnas.1608576114, PMID: 28028212 PMC5240698

[ref62] Liu Y. Xu X. H. Chen Q. Wang T. Deng C. Y. Song B. L.. (2013). Myosin Vb controls biogenesis of post-Golgi Rab10 carriers during axon development. Nat. Commun. 4:2005. doi: 10.1038/ncomms3005, PMID: 23770993

[ref63] Lowe M. Rabouille C. Nakamura N. Watson R. Jackman M. Jämsä E.. (1998). Cdc2 kinase directly phosphorylates the cis-Golgi matrix protein GM130 and is required for Golgi fragmentation in mitosis. Cell 94, 783–793. doi: 10.1016/S0092-8674(00)81737-7, PMID: 9753325

[ref64] Marín O. Valiente M. Ge X. Tsai L. H. (2010). Guiding neuronal cell migrations. Cold Spring Harb. Perspect. Biol. 2:a001834. doi: 10.1101/cshperspect.a001834, PMID: 20182622 PMC2828271

[ref65] Matsuki T. Chen J. Howell B. W. (2013). Acute inactivation of the serine-threonine kinase Stk25 disrupts neuronal migration. Neural Dev. 8:21. doi: 10.1186/1749-8104-8-21 24225308 PMC3874637

[ref66] Matsuki T. Matthews R. T. Cooper J. A. van der Brug M. P. Cookson M. R. Hardy J. A.. (2010). Reelin and stk25 have opposing roles in neuronal polarization and dendritic Golgi deployment. Cell 143, 826–836. doi: 10.1016/j.cell.2010.10.029, PMID: 21111240 PMC3033572

[ref67] Meseke M. Rosenberger G. Förster E. (2013). Reelin and the Cdc42/Rac1 guanine nucleotide exchange factor αPIX/Arhgef6 promote dendritic Golgi translocation in hippocampal neurons. Eur. J. Neurosci. 37, 1404–14012. doi: 10.1111/ejn.12153, PMID: 23406282

[ref68] Miao S. Chen R. Ye J. Tan G. H. Li S. Zhang J.. (2013). The Angelman syndrome protein Ube3a is required for polarized dendrite morphogenesis in pyramidal neurons. J. Neurosci. 33, 327–333. doi: 10.1523/JNEUROSCI.2509-12.2013, PMID: 23283345 PMC6618628

[ref69] Mikhaylova M. Bera S. Kobler O. Frischknecht R. Kreutz M. R. (2016). A Dendritic Golgi Satellite between ERGIC and retromer. Cell Rep. 14, 189–199. doi: 10.1016/j.celrep.2015.12.024, PMID: 26748700

[ref70] Mizuno H. Ikezoe K. Nakazawa S. Sato T. Kitamura K. Iwasato T. (2018). Patchwork-type spontaneous activity in neonatal barrel cortex layer 4 transmitted via thalamocortical projections. Cell Rep. 22, 123–135. doi: 10.1016/j.celrep.2017.12.012, PMID: 29298415

[ref71] Mohamed N. V. Desjardins A. Leclerc N. (2017). Tau secretion is correlated to an increase of Golgi dynamics. PLoS One 12:e0178288. doi: 10.1371/journal.pone.0178288, PMID: 28552936 PMC5446162

[ref72] Nakagawa N. Iwasato T. (2023). Golgi polarity shift instructs dendritic refinement in the neonatal cortex by mediating NMDA receptor signaling. Cell Rep. 42:112843. doi: 10.1016/j.celrep.2023.112843, PMID: 37516101

[ref73] Nakagawa N. Iwasato T. (2024). Activity-dependent dendrite patterning in the postnatal barrel cortex. Front. Neural Circuits 18:1409993. doi: 10.3389/fncir.2024.1409993, PMID: 38827189 PMC11140076

[ref74] Nakagawa N. Yagi H. Kato K. Takematsu H. Oka S. (2015). Ectopic clustering of Cajal-Retzius and subplate cells is an initial pathological feature in Pomgnt2-knockout mice, a model of dystroglycanopathy. Sci. Rep. 5:11163. doi: 10.1038/srep11163 26060116 PMC4461912

[ref75] Nakano A. (2022). The Golgi apparatus and its next-door neighbors. Front. Cell Dev. Biol. 10:884360. doi: 10.3389/fcell.2022.884360, PMID: 35573670 PMC9096111

[ref76] Nakazawa S. Yoshimura Y. Takagi M. Mizuno H. Iwasato T. (2020). Developmental phase transitions in spatial organization of spontaneous activity in postnatal barrel cortex layer 4. J. Neurosci. 40, 7637–7650. doi: 10.1523/JNEUROSCI.1116-20.2020, PMID: 32887743 PMC7531556

[ref77] Nichols A. J. Olson E. C. (2010). Reelin promotes neuronal orientation and dendritogenesis during preplate splitting. Cereb. Cortex 20, 2213–2223. doi: 10.1093/cercor/bhp303, PMID: 20064940 PMC2950812

[ref78] O’Dell R. S. Ustine C. J. Cameron D. A. Lawless S. M. Williams R. M. Zipfel W. R.. (2012). Layer 6 cortical neurons require Reelin-Dab1 signaling for cellular orientation, Golgi deployment, and directed neurite growth into the marginal zone. Neural Dev. 7:25. doi: 10.1186/1749-8104-7-25, PMID: 22770513 PMC3466444

[ref79] O’Donnell M. Chance R. K. Bashaw G. J. (2009). Axon growth and guidance: receptor regulation and signal transduction. Annu. Rev. Neurosci. 32, 383–412. doi: 10.1146/annurev.neuro.051508.135614, PMID: 19400716 PMC4765433

[ref80] Ogawa M. Miyata T. Nakajima K. Yagyu K. Seike M. Ikenaka K.. (1995). The reeler gene-associated antigen on Cajal-Retzius neurons is a crucial molecule for laminar organization of cortical neurons. Neuron 14, 899–912. doi: 10.1016/0896-6273(95)90329-1, PMID: 7748558

[ref81] Ori-McKenney K. M. Jan L. Y. Jan Y. N. (2012). Golgi outposts shape dendrite morphology by functioning as sites of acentrosomal microtubule nucleation in neurons. Neuron 76, 921–930. doi: 10.1016/j.neuron.2012.10.008, PMID: 23217741 PMC3523279

[ref82] Osen-Sand A. Catsicas M. Staple J. K. Jones K. A. Ayala G. Knowles J.. (1993). Inhibition of axonal growth by SNAP-25 antisense oligonucleotides in vitro and in vivo. Nature 364, 445–448. doi: 10.1038/364445a0, PMID: 8332215

[ref83] Pfenninger K. H. (2009). Plasma membrane expansion: a neuron's herculean task. Nat. Rev. Neurosci. 10, 251–621. doi: 10.1038/nrn2593, PMID: 19259102

[ref84] Pfenninger K. H. Laurino L. Peretti D. Wang X. Rosso S. Morfini G.. (2003). Regulation of membrane expansion at the nerve growth cone. J. Cell Sci. 116, 1209–1217. doi: 10.1242/jcs.00285, PMID: 12615964

[ref85] Powell S. K. Rivas R. J. Rodriguez-Boulan E. Hatten M. E. (1997). Development of polarity in cerebellar granule neurons. J. Neurobiol. 32, 223–236. doi: 10.1002/(SICI)1097-4695(199702)32:2<223::AID-NEU7>3.0.CO;2-A, PMID: 9032664

[ref86] Puthenveedu M. A. Bachert C. Puri S. Lanni F. Linstedt A. D. (2006). GM130 and GRASP65-dependent lateral cisternal fusion allows uniform Golgi-enzyme distribution. Nat. Cell Biol. 8, 238–248. doi: 10.1038/ncb1366, PMID: 16489344

[ref87] Quassollo G. Wojnacki J. Salas D. A. Gastaldi L. Marzolo M. P. Conde C.. (2015). A RhoA signaling pathway regulates dendritic Golgi outpost formation. Curr. Biol. 25, 971–982. doi: 10.1016/j.cub.2015.01.075, PMID: 25802147

[ref88] Rao S. Kirschen G. W. Szczurkowska J. Di Antonio A. Wang J. Ge S.. (2018). Repositioning of somatic Golgi apparatus is essential for the dendritic establishment of adult-born hippocampal neurons. J. Neurosci. 38, 631–647. doi: 10.1523/JNEUROSCI.1217-17.2017, PMID: 29217690 PMC5777113

[ref89] Ravichandran Y. Goud B. Manneville J. B. (2020). The Golgi apparatus and cell polarity: roles of the cytoskeleton, the Golgi matrix, and Golgi membranes. Curr. Opin. Cell Biol. 62, 104–113. doi: 10.1016/j.ceb.2019.10.003, PMID: 31751898

[ref90] Rice D. S. Curran T. (2001). Role of the reelin signaling pathway in central nervous system development. Annu. Rev. Neurosci. 24, 1005–1039. doi: 10.1146/annurev.neuro.24.1.1005, PMID: 11520926

[ref91] Rosso S. Bollati F. Bisbal M. Peretti D. Sumi T. Nakamura T.. (2004). LIMK1 regulates Golgi dynamics, traffic of Golgi-derived vesicles, and process extension in primary cultured neurons. Mol. Biol. Cell 15, 3433–3449. doi: 10.1091/mbc.e03-05-0328, PMID: 15090620 PMC452595

[ref92] Saraste J. Marie M. (2018). Intermediate compartment (IC): from pre-Golgi vacuoles to a semi-autonomous membrane system. Histochem. Cell Biol. 150, 407–430. doi: 10.1007/s00418-018-1717-2, PMID: 30173361 PMC6182704

[ref93] Shen K. Cowan C. W. (2010). Guidance molecules in synapse formation and plasticity. Cold Spring Harbor Persp. Biol. 2:a001842. doi: 10.1101/cshperspect.a001842, PMID: 20452946 PMC2845208

[ref94] Skupien-Jaroszek A. Szczepankiewicz A. A. Rysz A. Marchel A. Matyja E. Grajkowska W.. (2023). Morphological alterations of the neuronal Golgi apparatus upon seizures. Neuropathol. Appl. Neurobiol. 49:e12940. doi: 10.1111/nan.12940, PMID: 37771048

[ref95] Stafstrom C. E. Carmant L. (2015). Seizures and epilepsy: an overview for neuroscientists. Cold Spring Harb. Perspect. Med. 5:a022426. doi: 10.1101/cshperspect.a022426, PMID: 26033084 PMC4448698

[ref96] Stieber A. Gonatas J. O. Gonatas N. K. Louvard D. (1987). The Golgi apparatus-complex of neurons and astrocytes studied with an anti-organelle antibody. Brain Res. 408, 13–21. doi: 10.1016/0006-8993(87)90353-2, PMID: 3297246

[ref97] Stieber A. Mourelatos Z. Gonatas N. K. (1996). In Alzheimer's disease the Golgi apparatus of a population of neurons without neurofibrillary tangles is fragmented and atrophic. Am. J. Pathol. 148, 415–426, PMID: 8579105 PMC1861703

[ref98] Sun K. H. de Pablo Y. Vincent F. Johnson E. O. Chavers A. K. Shah K. (2008). Novel genetic tools reveal Cdk5's major role in Golgi fragmentation in Alzheimer's disease. Mol. Biol. Cell 19, 3052–3069. doi: 10.1091/mbc.e07-11-1106 18480410 PMC2441653

[ref99] Takeo Y. H. Yuzaki M. (2021). “Purkinje cell dendrites: the time-tested icon in histology” in Cerebellum as a CNS hub. eds. Mizusawa H. Kakei S. (Cham: Springer), 145–167.

[ref100] Tanabe K. Kani S. Shimizu T. Bae Y. K. Abe T. Hibi M. (2010). Atypical protein kinase C regulates primary dendrite specification of cerebellar Purkinje cells by localizing Golgi apparatus. J. Neurosci. 30, 16983–16992. doi: 10.1523/JNEUROSCI.3352-10.2010, PMID: 21159968 PMC6634915

[ref101] Targa Dias Anastacio H. Matosin N. Ooi L. (2022). Neuronal hyperexcitability in Alzheimer's disease: what are the drivers behind this aberrant phenotype? Transl. Psychiatry 12:257. doi: 10.1038/s41398-022-02024-7, PMID: 35732622 PMC9217953

[ref102] Thayer D. A. Jan Y. N. Jan L. Y. (2013). Increased neuronal activity fragments the Golgi complex. Proc. Natl. Acad. Sci. U. S. A. 110, 1482–1487. doi: 10.1073/pnas.1220978110, PMID: 23297202 PMC3557034

[ref103] Tomás M. Martínez-Alonso E. Martínez-Martínez N. Cara-Esteban M. Martínez-Menárguez J. A. (2021). Fragmentation of the Golgi complex of dopaminergic neurons in human substantia nigra: new cytopathological findings in Parkinson's disease. Histol. Histopathol. 36, 47–60. doi: 10.14670/HH-18-270, PMID: 33078843

[ref104] Tremblay R. Lee S. Rudy B. (2016). GABAergic interneurons in the neocortex: from cellular properties to circuits. Neuron 91, 260–292. doi: 10.1016/j.neuron.2016.06.033, PMID: 27477017 PMC4980915

[ref105] Tzilivaki A. Tukker J. J. Maier N. Poirazi P. Sammons R. P. Schmitz D. (2023). Hippocampal GABAergic interneurons and memory. Neuron 111, 3154–3175. doi: 10.1016/j.neuron.2023.06.016, PMID: 37467748 PMC10593603

[ref106] Valenzuela J. I. Perez F. (2015). Diversifying the secretory routes in neurons. Front. Neurosci. 9:358. doi: 10.3389/fnins.2015.00358, PMID: 26500481 PMC4595659

[ref107] Valiente M. Marín O. (2010). Neuronal migration mechanisms in development and disease. Curr. Opin. Neurobiol. 20, 68–78. doi: 10.1016/j.conb.2009.12.003, PMID: 20053546

[ref108] Wamsley B. Fishell G. (2017). Genetic and activity-dependent mechanisms underlying interneuron diversity. Nat. Rev. Neurosci. 18, 299–309. doi: 10.1038/nrn.2017.30, PMID: 28381833

[ref109] Wang J. Fourriere L. Gleeson P. A. (2020). Local secretory trafficking pathways in neurons and the role of dendritic Golgi outposts in different cell models. Front. Mol. Neurosci. 13:597391. doi: 10.3389/fnmol.2020.597391, PMID: 33324160 PMC7726432

[ref110] Wang T. Liu Y. Xu X. H. Deng C. Y. Wu K. Y. Zhu J.. (2011). Lgl1 activation of rab10 promotes axonal membrane trafficking underlying neuronal polarization. Dev. Cell 21, 431–444. doi: 10.1016/j.devcel.2011.07.007, PMID: 21856246

[ref111] Will L. Portegies S. van Schelt J. van Luyk M. Jaarsma D. Hoogenraad C. C. (2019). Dynein activating adaptor BICD2 controls radial migration of upper-layer cortical neurons in vivo. Acta Neuropathol. Commun. 7:162. doi: 10.1186/s40478-019-0827-y 31655624 PMC6815425

[ref112] Wong R. O. Ghosh A. (2002). Activity-dependent regulation of dendritic growth and patterning. Nat. Rev. Neurosci. 3, 803–812. doi: 10.1038/nrn941, PMID: 12360324

[ref113] Wu J. Akhmanova A. (2017). Microtubule-Organizing Centers. Annu. Rev. Cell Dev. Biol. 33, 51–75. doi: 10.1146/annurev-cellbio-100616-060615 28645217

[ref114] Wu Y. K. Fujishima K. Kengaku M. (2015). Differentiation of apical and basal dendrites in pyramidal cells and granule cells in dissociated hippocampal cultures. PLoS One 10:e0118482. doi: 10.1371/journal.pone.0118482, PMID: 25705877 PMC4338060

[ref115] Yadav S. Linstedt A. D. (2011). Golgi positioning. Cold Spring Harb. Perspect. Biol. 3:a005322. doi: 10.1101/cshperspect.a005322, PMID: 21504874 PMC3101843

[ref116] Yanagida M. Miyoshi R. Toyokuni R. Zhu Y. Murakami F. (2012). Dynamics of the leading process, nucleus, and Golgi apparatus of migrating cortical interneurons in living mouse embryos. Proc. Natl. Acad. Sci. U. S. A. 109, 16737–16742. doi: 10.1073/pnas.1209166109 23010922 PMC3478636

[ref117] Ye B. Zhang Y. Song W. Younger S. H. Jan L. Y. Jan Y. N. (2007). Growing dendrites and axons differ in their reliance on the secretory pathway. Cell 130, 717–729. doi: 10.1016/j.cell.2007.06.032, PMID: 17719548 PMC2020851

[ref118] Yin D. M. Huang Y. H. Zhu Y. B. Wang Y. (2008). Both the establishment and maintenance of neuronal polarity require the activity of protein kinase D in the Golgi apparatus. J. Neurosci. 28, 8832–8843. doi: 10.1523/JNEUROSCI.1291-08.2008, PMID: 18753385 PMC6670825

[ref119] Zhang Y. Kunii M. Taniguchi M. Yoshimura S. I. Harada A. (2024). Rab6-mediated polarized transport of synaptic vesicle precursors is essential for the establishment of neuronal polarity and brain formation. J. Neurosci. 44:e2334232024. doi: 10.1523/JNEUROSCI.2334-23.2024, PMID: 38830762 PMC11223463

[ref120] Zmuda J. F. Rivas R. J. (1998). The Golgi apparatus and the centrosome are localized to the sites of newly emerging axons in cerebellar granule neurons *in vitro*. Cell Motil. Cytoskeleton 41, 18–38. doi: 10.1002/(SICI)1097-0169(1998)41:1<18::AID-CM2>3.0.CO;2-B, PMID: 9744296

